# Fluorescence Lifetime Phasor Analysis of the Decamer–Dimer Equilibrium of Human Peroxiredoxin 1

**DOI:** 10.3390/ijms23095260

**Published:** 2022-05-09

**Authors:** Sebastián F. Villar, Joaquín Dalla-Rizza, Matías N. Möller, Gerardo Ferrer-Sueta, Leonel Malacrida, David M. Jameson, Ana Denicola

**Affiliations:** 1 Laboratorio de Fisicoquímica Biológica, Instituto de Química Biológica, Facultad de Ciencias, Universidad de la República, Montevideo 11400, Uruguay; s.villarrodriguez@fcien.edu.uy (S.F.V.); joaquindallari@gmail.com (J.D.-R.); mmoller@fcien.edu.uy (M.N.M.); gferrer@fcien.edu.uy (G.F.-S.); 2 Centro de Investigaciones Biomédicas (CEINBIO), Universidad de la República, Montevideo 11800, Uruguay; 3 Advanced Bioimaging Unit, Institut Pasteur de Montevideo, Montevideo 11400, Uruguay; lmalacrida@pasteur.edu.uy; 4 Departamento de Fisiopatología, Hospital de Clínicas, Universidad de la República, Montevideo 11600, Uruguay; 5 Department of Cell and Molecular Biology, University of Hawaii at Manoa, Honolulu, HI 96822, USA

**Keywords:** peroxiredoxin 1, protein oligomerization, lifetime phasors, spectral phasors, tryptophan fluorescence, dissociation constant

## Abstract

Protein self-assembly is a common feature in biology and is often required for a myriad of fundamental processes, such as enzyme activity, signal transduction, and transport of solutes across membranes, among others. There are several techniques to find and assess homo-oligomer formation in proteins. Naturally, all these methods have their limitations, meaning that at least two or more different approaches are needed to characterize a case study. Herein, we present a new method to study protein associations using intrinsic fluorescence lifetime with phasors. In this case, the method is applied to determine the equilibrium dissociation constant (K_D_) of human peroxiredoxin 1 (hPrx1), an efficient cysteine-dependent peroxidase, that has a quaternary structure comprised of five head-to-tail homodimers non-covalently arranged in a decamer. The hPrx1 oligomeric state not only affects its activity but also its association with other proteins. The excited state lifetime of hPrx1 has distinct values at high and low concentrations, suggesting the presence of two different species. Phasor analysis of hPrx1 emission lifetime allowed for the identification and quantification of hPrx1 decamers, dimers, and their mixture at diverse protein concentrations. Using phasor algebra, we calculated the fraction of hPrx1 decamers at different concentrations and obtained K_D_ (1.1 × 10^−24^ M^4^) and C_0.5_ (1.36 μM) values for the decamer–dimer equilibrium. The results were validated and compared with size exclusion chromatography. In addition, spectral phasors provided similar results despite the small differences in emission spectra as a function of hPrx1 concentration. The phasor approach was shown to be a highly sensitive and quantitative method to assess protein oligomerization and an attractive addition to the biophysicist’s toolkit.

## 1. Introduction

Proteins in living organisms are often composed of multiple subunits, which may be identical (homo-oligomers) or different (hetero-oligomers). Homo-oligomers are prevalent in nature (30–50% proteome) and play important roles in biology such as modulation of enzyme activities, cooperativity, gene expression, and stability [1,2].

There are different techniques to characterize the self-association of proteins, including size exclusion chromatography (SEC), analytical ultracentrifugation, light scattering techniques, isothermal titration calorimetry (ITC), fluorescence techniques, and mass spectrometry [2,3]. Information on stoichiometry and/or binding affinities can be obtained with one or a combination of several of these techniques [4]. Unfortunately, there are not many suitable methods to study protein oligomerization using steady-state protein concentrations. Usually, the ones available are not very sensitive or require a considerable amount of sample (DLS, SAXS, ITC). 

The intrinsic fluorescence of proteins, dominated by the aromatic tryptophan residue (W), is affected by its surroundings including solvent accessibility, and the presence of particular amino acid residues that can modulate the maximum wavelength of emission, the quantum yield, and the lifetime [5,6]. Tryptophan emission has proven to be a useful reporter of protein structural changes because it is sensitive to perturbations in its molecular environment [7]. Therefore, intrinsic protein fluorescence offers a tag-free approach to study many biophysical properties of proteins, including oligomerization. 

Among the intrinsic protein fluorescence techniques available to study protein associations, time-resolved fluorescence is the least explored. One of the reasons is the complex nature of protein tryptophan emission decay, which generally cannot be fit to a single exponential term even in proteins with only one tryptophan residue [6,8,9,10]. Nevertheless, the use of phasors simplifies the visualization and processing of time-resolved fluorescence data. This approach has the advantage that it compiles all the complexity of the sample’s fluorescence emission lifetime into one point within a 2D plot, called the phasor plot. This approach has been thoroughly described elsewhere [11,12], and has been mainly used on live cells fluorescence microscopy studies [13], and less extensively in vitro [14,15,16]. 

Fluorescence lifetime phasor plots can be constructed using either time-domain or frequency-domain data. In this study, frequency domain data were used, and thus we focused on this approach. Briefly, from frequency domain fluorescence lifetime measurements, two values, termed G and S, are calculated from the phase delay (φ) and the modulation ratio (M) at a particular light modulation frequency as follows:

G = M cosφ(1)

S = M sinφ (2)

**Figure 1:**
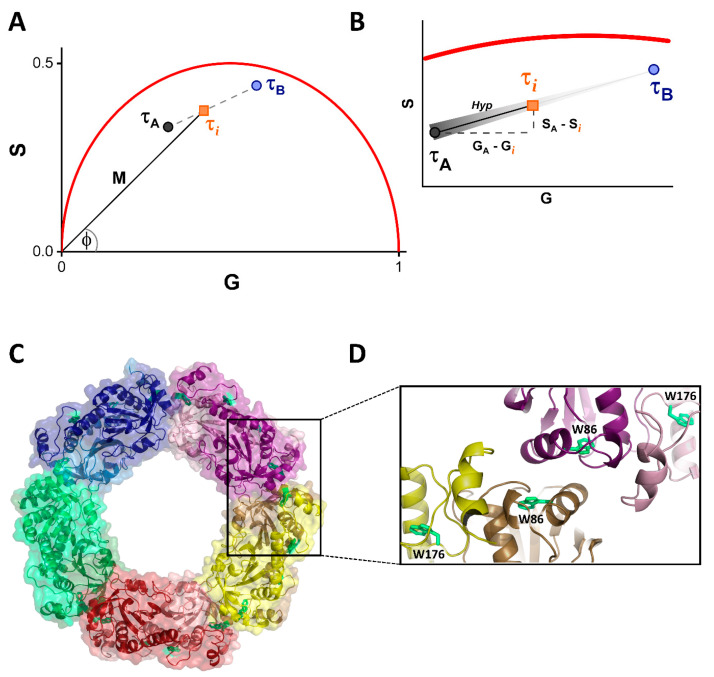
**Fluorescence lifetime phasors approach and human peroxiredoxin 1 structure.** (**A**) Phasor plot of two multiexponential decays (**A**,**B**) and a mixture of them (*i*). The universal circle is delimited by the red curve; τ_A_ and τ_B_ are indicated as black and blue circles, respectively; and τ*_i_* is represented as an orange square. The phasor of τ*_i_* is drawn from the origin, with module M and angle φ. All possible combinations of A and B fall on the segment (dashed line) between the individual components. (**B**) Zoom in of the A-B segment and determination of the fraction of A in *i*. The fading black shade indicates the amount of A along the segment. The A-*i* distance (Hyp) is calculated using the Pythagorean theorem as indicated by the dashed lines. The A-B distance is needed in order to calculate the fraction of components. (**C**) Structure of decameric hPrx1 (homology model, template PDB: 2Z9S). The homodimers forming the decamer are shown in different colors. The monomers of each homodimer are represented as different shades of the same color. (**D**) The dimer-dimer interface that separates upon dissociation. Tryptophan residues are represented as green sticks. The residue W86 is inside the interface, whereas W176 is outside.

G and S are the equivalent of x and y values of a cartesian coordinate axis. The phasor (“phase vector”) is constructed from the origin of the axis using G and S coordinates as shown in Figure 1A, where the modulation and angle of the phasor are represented by M and φ, respectively (τ*_i_* phasor). The red line delimiting a semicircle (called a “universal circle”) is the curve where all the fluorophores with monoexponential decays fall, whereas multiexponential decays will fall inside the universal circle [12]. All these parameters and rules of the phasor plot for time-resolved fluorescence were first described and developed by Gregorio Weber [17]. 

One remarkable property of the phasor plot is that it easily allows one to calculate the exact fraction of individual fluorophores within a mixture. In the situation depicted in Figure 1A, two fluorophores with multiexponential decays (τ_A_ and τ_B_) are represented as points inside the universal circle. A mixture of A and B called *i* lies on the line segment between them. Given the algebraic properties of phasors, *i* is the linear combination of the individual lifetime components A and B, so all combinations of both components will fall on the line segment A-B (dashed line). Thus, the fraction of each component can be calculated as the ratio of the distance between *i* and either end of the A-B segment and the total length of the segment (Equation (7)). In this case, the distance between A and *i* is calculated as the hypotenuse (Hyp) of a triangle (Figure 1B), using the Pythagorean theorem [16]. This distance is inversely proportional to the fraction of A (fading black shade indicating amount of A along the segment, Figure 1B), so if *i* reaches B, the amount of A is zero.

This scenario can be translated to the study of protein self-associations, especially in the case of homo-oligomers that have a binary behavior. For example, in the case of monomer–dimer associations, if the points A and B represent the individual species with their characteristic τ values, *i* describes the distribution of monomers and dimers at any point of the equilibrium (Figure 1A,B). Therefore, this method can quantify the amount of each oligomeric species present in a certain steady-state condition and determine the dissociation/association constant. We should note that the fractions in the phasor plots correspond to the fractional intensities of the emitted photons from each species. If there is a change in the quantum yield of the two species, then this change must be taken into account to calculate the molar fractions involved.

Herein, we used fluorescence lifetime phasors to study the dissociation of decameric human peroxiredoxin 1 (Figure 1C). Peroxiredoxins (Prxs) are a ubiquitous family of multifunctional proteins that are mostly recognized for their antioxidant role, being able to reduce a wide range of hydroperoxides using a specialized cysteine residue [17,18]. Additionally, these enzymes possess holdase and/or chaperone function [19,20,21] and are involved in peroxide dependent signaling events related to cell physiology [19,22,23,24]. Prxs from the Prx1 subfamily have two active site cysteine residues that are used to reduce hydrogen peroxide (H_2_O_2_) at the expense of the thioredoxin/thioredoxin-reductase/NADPH system [25,26]. These proteins are obligated homodimers able to form ring-shaped decamers or dodecamers that can further assemble into stacked rings [27,28,29]. In particular, human Prx1 (hPrx1) is either a decamer or a dimer that contains two tryptophan residues (W86 and 176) that are shown to be sensitive to structural changes induced by the dissociation of the decamer into dimers (Figure 1C,D). 

## 2. Materials

### 2.1. Human Prx1

Recombinant hPrx1 was expressed in *E.coli* BL21(DE3) cells and purified from bacterial lysates, as previously described [18]. Prior to all experiments and measurements, hPrx1 was reduced with 10 mM dithiothreitol (DTT) in PBS buffer (137 mM NaCl, 2.7 mM KCl, 10 mM Na_2_HPO_4_, 1.8 mM KH_2_PO_4_) pH 7.4 for 30 min to 1 h, at room temperature and with orbital agitation. The excess of reductant was removed with a PD-10 column (cytiva) and, if needed, the eluted protein was concentrated with Vivaspin 6 centricons of 10 kDa MWCO (cytiva). Reduced hPrx1 was quantified by absorbance at 280 nm, using ε_280_ = 18,450 M^−1^cm^−1^. Unless explicitly indicated, the protein concentration of hPrx1 will refer to monomer.

### 2.2. Size Exclusion Chromatography 

Size exclusion chromatography was performed in a HPLC instrument (Agilent 1260) with a Superdex 200 5/150 GL column (cytiva). The runs were performed in PBS pH 7.4 supplemented with 1 mM DTT (to prevent protein oxidation), with a flow rate of 0.4 mL/min at room temperature (22–25 °C). Samples were injected in a volume of 100 µL. For each run, fluorescence emission was recorded at 340 nm using an excitation of 280 nm. Photo multiplier tube (PMT) gain was increased for low protein concentrations. All the chromatograms were normalized. 

The decamer fraction (X_decamer_) at each initial hPrx1 concentration was calculated considering the area under the curve (AUC) for the decamer (elution time = 3.9 min), the dimer (t = 5.2 min), and their sum (Equation (3)).

(3) $$X_{\mathrm{decamer}}=\frac{{\mathrm{AUC}}_{\mathrm{decamer}}}{{\mathrm{AUC}}_{\mathrm{decamer}}+{\mathrm{AUC}}_{\mathrm{dimer}}}$$

The AUC for both species was obtained as follows: a specific Δt for the decamer (3.0–4.3 min) and the dimer (4.9–6.5 min) was established from the chromatograms where the decamer or the dimer were the only oligomeric forms. Then, all the chromatograms were integrated using the established Δt values, and the AUC of each species was determined at different hPrx1 concentrations. 

### 2.3. Fluorescence Emission Spectra

Emission spectra of hPrx1 from 300 to 500 nm were recorded with a ChronosFD spectrofluorometer (ISS) in PBS supplemented with 1 mM DTT, at 25 °C, using excitation wavelength (λ_exc_) of 280 nm. Slits of 8 nm and 16 nm were used in the excitation and emission paths, respectively. The samples were prepared outside the cuvette and left to stabilize at room temperature for 25 min. Additionally, each sample was incubated for 5 min inside the thermostated cuvette holder before measurement.

The spectral center of mass (CM) can be calculated using the following equation:

(4) $$\mathrm{CM}=\frac{\sum_{i}{\mathrm{IF}}_{i}\lambda_{i}}{\sum_{i}{\mathrm{IF}}_{i}}$$

where IF and λ are the intensity of fluorescence and the wavelength, respectively. In addition, the emission spectra were analyzed using phasors, calculating the G and S values of the spectral phasor as previously described [30,31]:

(5) $$G=\frac{\sum_{i}{\mathrm{IF}}_{i}\cos\frac{2\pi n\left(\lambda_{i}-\lambda_{0}\right)}{L}}{\;\sum_{i}{\mathrm{IF}}_{i}}$$

(6) $$S=\frac{\sum_{i}{\mathrm{IF}}_{i}\sin\frac{2\pi n\left(\lambda_{i}-\lambda_{0}\right)}{L}}{\;\sum_{i}{\mathrm{IF}}_{i}}$$

where λ_0_ is the initial wavelength of the spectrum (300 nm), L is the length of the spectrum (200 nm), and n is the harmonic value (in this case we used the first harmonic, n = 1). 

### 2.4. Fluorescence Lifetime Analog Frequency Domain Measurements

Lifetime measurements were performed in the frequency domain with the ISS ChronosFD instrument. The light source was a 284 nm LED, modulated by frequency synthesizers within a range of 10 to 200 MHz. A 280 nm bandpass filter was used in the excitation path. Fluorescence emission was collected through a WG320 long pass filter. N-Acetyl-L-tryptophanamide (NATA) was used as a reference (τ = 2.8 ns at 25 °C); Abs_280nm_ values were between 0.15 and 0.18. At a frequency of 10 MHz, the intensities of the sample and the reference were equalized using neutral density filters in the excitation and/or the emission path. Measurements were performed in PBS supplemented with 1 mM DTT to ensure all the hPrx1 is in the reduced state. Since reduction of the enzyme with DTT is slow, the samples were prepared outside the cuvette and left at room temperature for 25 min. Additionally, each sample was incubated for 5 min inside the thermostated cuvette holder before the measurement. Absolute lifetime measurements were performed with excitation polarizers placed vertically and emission polarizers placed at the “magic angle” (54.7°). To obtain protein dissociation curves from fluorescence lifetime phasor plots, the lifetime measurements were performed without polarizers and with a time base of 3 s.

### 2.5. Fluorescence Lifetime Phasor Plot Analysis

In this case, X_decamer_ was calculated assuming the following: (a) the decamer and the dimer of hPrx1 are the only significant fluorescent species in the sample; (b) the lifetime of any mixture of decamers and dimers is a linear combination of their lifetime components; (c) the ratio of the linear combination determines the fraction of decamers and dimers.

So, we consider the phasor points of the decamer and the dimer as the endpoints of a line segment, and all the points that lay on this line represent different fractions of both oligomeric forms, similar to the situation described in Figure 1A,B. Taking this into account, X_decamer_ at each hPrx1 concentration is calculated as

(7) $$X_{\mathrm{decamer}}=1-\frac{\sqrt{{\left(G_{A}-G_{i}\right)}^{2}+{\left(S_{A}-S_{i}\right)}^{2}}}{\sqrt{{\left(G_{A}-G_{B}\right)}^{2}+{\left(S_{A}-S_{B}\right)}^{2}}}$$

where (G_A_,S_A_) is the phasor at the highest hPrx1 concentration that exclusively represents the decamer (X_decamer_ = 1); (G_B_,S_B_) is the endpoint phasor of the line segment that exclusively represents the dimer, where X_decamer_ = 0; and (G*i*,S*i*,) is any phasor in between the former two. In Equation (7), the distances between phasors are calculated as the coordinate subtraction of the endpoints of a hypotenuse using the Pythagorean theorem. Only the distances alongside the A-B segment were considered to build the dissociation curves.

### 2.6. Fitting Model

The dissociation of a decamer to five dimers, as occurs in these peroxiredoxins, can be expressed by the following K_D_:

(8) decamer↔5 dimer

(9) $$K_{D}=\frac{{\left[\mathrm{dimer}\right]}^{5}}{\left[\mathrm{decamer}\right]}$$

X_decamer_ can be described in terms of the dimer concentration of hPrx1 as follows:

(10) $$X_{\mathrm{decamer}}=\frac{5\left[\mathrm{decamer}\right]}{5\left[\mathrm{decamer}\right]+\left[\mathrm{dimer}\right]}$$

From the dissociation equilibrium (Equation (8)), we can define [decamer] as a function of [dimer]:

(11) $$\left[\mathrm{decamer}\right]=\frac{{\left[\mathrm{dimer}\right]}^{5}}{K_{D}}$$

If we substitute the [decamer] of Equation (10) with Equation (11) and apply some basic algebra, we can obtain the following expression:

(12) $$X_{\mathrm{decamer}}=\frac{{\left[\mathrm{dimer}\right]}^{4}}{{\left[\mathrm{dimer}\right]}^{4}+\frac{1}{\;5}K_{D}}$$

Equation (13) indicates the value of hPrx1 monomer concentration where subunits are equally distributed between dimers and decamers (C_0.5_):

(13) $$C_{0.5}=2\sqrt[{4}]{\frac{K_{D}}{5}}$$

This C_0.5_ is a more helpful descriptor of the equilibrium than the K_D_ value since the latter has units of M^4^, making it rather complicated to interpret and somewhat unrelatable. 

## 3. Results

### 3.1. Fluorescence Properties of hPrx1 and Their Dependance on Protein Concentration

First, we studied the fluorescence properties of hPrx1 to find which property was most sensitive to changes in protein concentration. 

**Figure 2:**
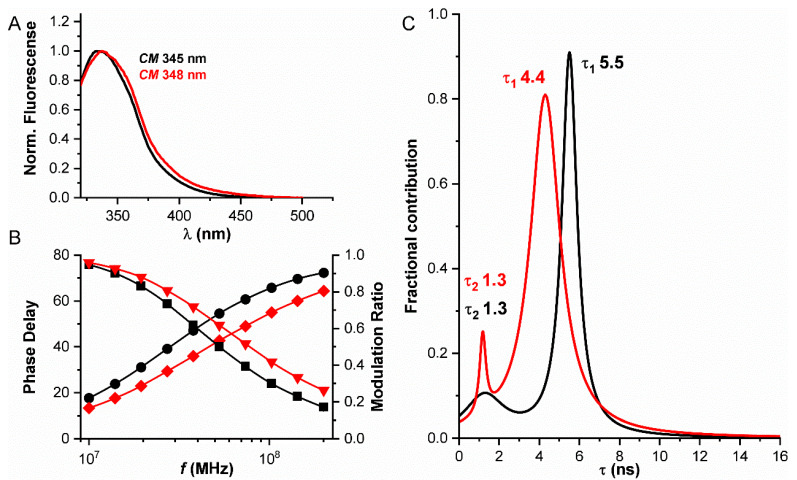
**Fluorescence properties of hPrx1 depend on its concentration.** Black and red traces correspond to 20 μM and 0.5 μM hPrx1, respectively. (**A**) Emission spectra of hPrx1 with λ_exc_ 280 nm; the spectral center of mass (CM) values are indicated. (**B**) Phase delay (circles and diamonds) and modulation ratio (squares and triangles) plots obtained from analog frequency domain lifetime measurements at different light modulation frequencies. (**C**) Lorentz distributions obtained from the fit of emission lifetimes. Lifetime values obtained from the Lorentzian fit are indicated.

The fluorescence emission spectra and lifetime of hPrx1 were measured at 20 μM and 0.5 µM (Figure 2). From the normalized emission spectra and their center of mass, we can see a difference between the two concentrations assayed, a 3 nm red shift in the emission at 0.5 µM relative to 20 µM (Figure 2A). This red shift can be preliminarily related to a change in the environment of tryptophan residues as a result of the dissociation from decamers to dimers. 

Analog frequency domain determinations of the emission lifetime of hPrx1 indicated the presence of two components (τ_1_ and τ_2_), after the results were fitted to a Lorentz distribution. This fitting model was chosen because of its low χ^2^ (≈0.46) value compared to other models (discrete ≈ 2.53, and Gaussian ≈ 1.1). A decrease in the principal lifetime component, τ_1_, from 20 μM to 0.5 μM hPrx1, from 5.5 to 4.4 ns was observed, while the second component, τ_2_, did not change (1.3 ns) (Figure 2B). Furthermore, the shape of the distributions was different in each case, as we observed a change in their widths. Although the number of components found matches the number of tryptophan residues in hPrx1, it is not necessarily correct to assign τ values to a specific tryptophan residue, since proteins have a dynamic behavior and explore different conformations that may alter tryptophan emission, resulting in complex emission lifetimes [6,9]. It is clear that there is a change in the fluorescence emission lifetime at the two concentrations assayed, and this change is more pronounced than the one observed in the emission spectra of hPrx1 (Figure 2C). 

### 3.2. Dissociation of hPrx1 Followed by Fluorescence Lifetime Phasors

As is usually the case in protein tryptophan emission, the intrinsic fluorescence decay of hPrx1 is complex, and even though a significant difference in tryptophan lifetimes was observed at the two concentrations assayed, fitted lifetime parameters do not provide a facile way to study protein associations. The phasor approach has the advantage that it essentially compiles all the emission lifetime complexity into a point and simplifies the analysis while retaining valuable information [13]. Thus, we used the phasor approach to quantitatively assess the decamer–dimer equilibrium of hPrx1.

**Figure 3:**
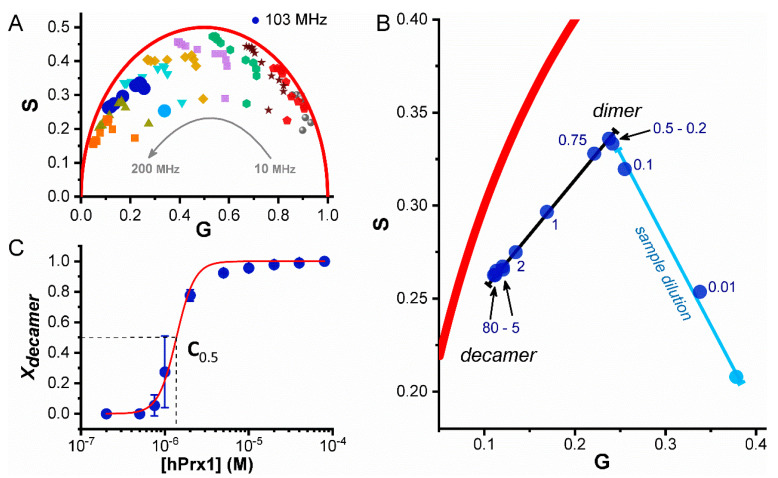
**Dissociation of hPrx1 followed by fluorescence lifetime phasors**. (**A**) Multifrequency domain (10–200 MHz) fluorescence lifetime measurements were performed at different hPrx1 concentrations (80–0.01 μM) and converted to phasor points. The colors represent different frequencies, and the multiplicity of points represent the different concentrations assayed. (**B**) Phasor points obtained at 103 MHz are shown. A black line connects phasor points obtained at high (80 μM) and low (0.2 μM) concentrations of hPrx1. Further dilution of hPrx1 led to signal loss and phasor displacement towards the buffer fluorescence (light blue line and point). The numbers indicate hPrx1 concentration (μM) at each point. (**C**) Data fitting to a decamer to dimer dissociation model. The fraction of decamer was calculated from the phasor coordinates using Equation (7), plotted against hPrx1 concentration, and fitted to the model (Equation (12)). Data are represented as the average ± standard deviation from three independent experiments.

The fluorescence lifetime was clearly sensitive to changes in the concentration of hPrx1. The oligomeric state of hPrx1 is easier to appreciate using the phasor approach (Figure 3). Different hPrx1 concentrations lead to changes in lifetime and the phasor points. A logarithmic frequency scan within a range of 10 to 200 MHz was performed for all protein concentrations in order to find a frequency at which the phasor points distribute best along a line segment (Figure 3A), and we chose 103 MHz as the frequency that best met our criterion and used these data for the phasor analysis. Figure 3 shows the data obtained and describes, step by step, how the dissociation curve is built using the phasor analysis. The black line in Figure 3B represents the oligomerization trajectory, from decamers (left end) to dimers (right end) at high and low hPrx1 concentrations, respectively. The accumulation of points at either end of the trajectory helped us to define specific regions for each oligomeric species. We detected that from 80 to 20 μM, the phasor points were tightly close to each other, and therefore we defined 80 μM as the starting point, representing exclusively the hPrx1 decamer. On the opposite end, phasor points from 0.75 to 0.2 μM, demonstrated the same behavior, and we defined 0.2 μM as the end point, which exclusively represents the dimer. The straight line connecting the phasor points suggests that the presence of intermediate oligomers is scarce [32], likely because the interconversion between decamers and dimers is fast or intermediates are not stable enough to be detected. Having defined the total trajectory, we were able to calculate X_decamer_ at all concentrations assayed between 80 μM and 0.2 μM hPrx1 using Equation (7). Plotting X_decamer_ against the hPrx1 concentration, we built the dissociation curve (Figure 3C) and performed the fitting to our model (Equation (12)). From the fit, we obtained a K_D_ of (1.1 ± 0.2) × 10^−24^ M^4^ (R-Square = 0.99), and using Equation (13), we calculated a C_0.5_ value of 1.36 ± 0.24 μM, which indicates the hPrx1 concentration where half of the monomers are forming decamers and the other half dimers. 

Interestingly, a second trajectory was identified in the phasor plot and consisted of the movement of low concentration points from the dimer zone to the background signal (Figure 3B, light blue point). We assigned the latter to the dilution of our sample and not to any other oligomeric transition (dimer to monomer), and thus the phasor points that fell on this new segment (0.1 and 0.01 μM) were not considered for the dissociation curve. At these protein concentrations, i.e., as the fluorescence from the protein decreased, the relative contribution of the background (buffer) to the overall signal started to increase; thus, the phasor points were pulled from the dimer region towards the background. We note that the background fluorescence issue is often observed in fluorescence lifetime microscopy (FLIM) studies on cells that may have significant autofluorescence. 

### 3.3. Emission Spectra Analysis Using Spectral Phasors 

**Figure 4:**
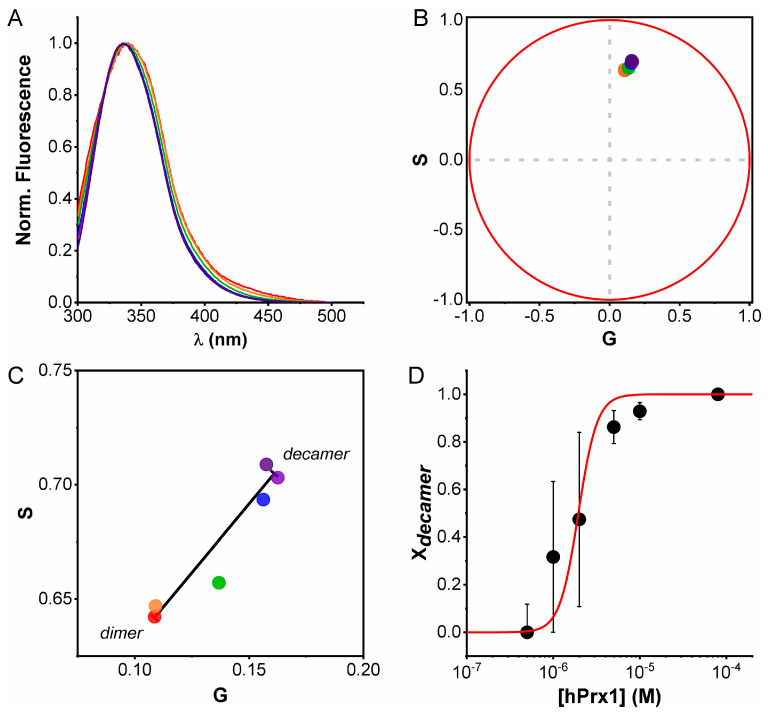
**Dissociation of hPrx1 followed by spectral phasors**. (**A**) Normalized emission spectra of hPrx1 (λ_exc_280 nm) at different concentrations (from red to dark violet: 0.5, 1, 2, 5, 10, and 80 µM). (**B**) Spectral phasor plot of the emission spectra in (**A**). (**C**) Phasor points for each emission spectrum are shown (zoom-in of (**B**)). The decamer–dimer transition is represented by the black line. (**D**) Plot of X_decamer_ vs. hPrx1 concentration and fitting to the dissociation model. Data are represented as the average ± standard deviation from three independent experiments.

Despite the small difference observed in hPrx1 emission spectra at high and low concentrations (Figure 2A), we wanted to know whether it was possible to extract information about the oligomerization equilibrium from spectral data. To serve this purpose, we decided to transform the spectra at different hPrx1 concentrations to the phasor space using spectral phasors (Equations (5) and (6)). Unlike spectral center of mass, spectral phasors provide a simplified and quantitative manner to analyze spectral data, very similar to lifetime phasors. Analysis of the emission spectra using spectral phasors enabled us to identify hPrx1 decamers and dimers separated along a line segment and intermediate concentrations falling in between (Figure 4B,C). Since we were in the phasor space, we used the same analysis we conducted with lifetime phasors (Equation (7)) and calculated X_decamer_ at the different hPrx1 concentrations. From the dissociation curve of Figure 4D, we obtained K_D_ and C_0.5_ values of (4.5 ± 2.3) × 10^−24^ M^4^ (R-Square = 0.89) and 1.95 ± 0.99 μM, respectively. Even though the resemblance with the lifetime phasors experiment was high, the dynamic range for decamers and dimers was much shorter for the spectral phasors, and the points near the transition carry more error.

### 3.4. Size Exclusion Chromatography Analysis 

Size exclusion chromatography (SEC) was used to validate the results obtained by fluorescence lifetime phasors. SEC is a classical method to study quaternary structure where the different oligomeric forms are separated according to their hydrodynamic radii. However, if dissociation is relatively fast, and protein concentration is low, the protein is further diluted during chromatography and will change its oligomeric state since the equilibrium is dynamic during the run. Thus, SEC should be taken as an approximation that may likely provide an overestimation of the protein C_0.5_. To avoid dilution problems as much as possible, a small column was used (void volume = 1.07 mL), and a high volume of protein at the desired concentration was injected (100 μL). To improve detection of small quantities of protein, the elution was followed by fluorescence, which allowed the detection of 0.3 pmoles of protein (3 nM injected, data not shown). 

**Figure 5:**
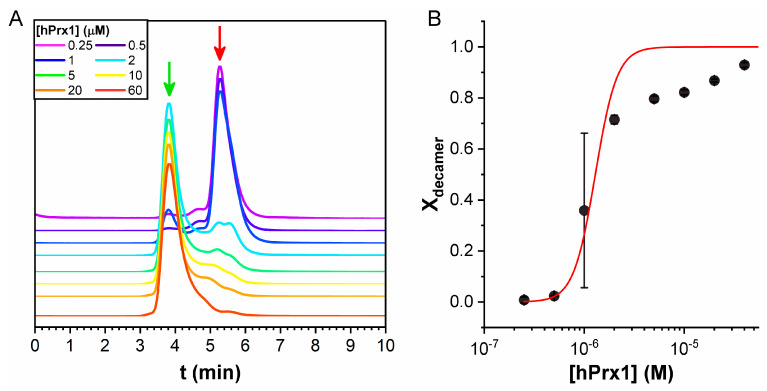
**Dissociation of hPrx1 studied by SEC**. (**A**) Chromatograms obtained at different initial hPrx1 concentrations, showing the decamer at 3.9 min (green arrow) and the dimer at 5.2 min (red arrow). (**B**) Dissociation curve of the calculated X_decamer_ from the area under the curves as a function of hPrx1 concentration. Data are represented as the average ± standard deviation from two independent experiments.

At high hPrx1 concentrations, a peak at 3.9 min appeared, corresponding to a mass of 270 kDa, consistent with the expected mass of the decamer (220 kDa), while at low hPrx1 concentrations, the main peak shifted to 5.2 min, corresponding to a mass of 42 kDa, consistent with the dimer (44 kDa) (Figure 5A). Area analysis of the chromatograms revealed that the peak concentrations were close to 35% of the injected concentration, except for 1 and 2 μM, in which the areas of both dimer and decamer peaks were significant. 

The dissociation curve was built with the calculated X_decamer_ obtained from the peak areas (Equation (3)) as a function of hPrx1 concentration. SEC results showed a K_D_ of (8.6 ± 4.6) × 10^−25^ M^4^ and C_0.5_ value of 1.28 ± 0.68 μM (Figure 5B). However, the SEC data (Figure 5B) did not fit very well to the model (R-Square = 0.89), and the differences were larger at concentrations above 2 μM. This result could be explained by the fact that in SEC, the protein concentration was not in a steady state; thus, the constant dilution of the protein during the run favored the dissociation.

Nevertheless, SEC results validated the initial estimate in the previous fluorescence experiments because the chromatography unambiguously showed that at 20 μM and 0.5 μM hPrx1, the predominant species were the decamer and the dimer, respectively. Furthermore, these data also support the conclusion that the observed changes in the fluorescent properties of hPrx1 are linked to its quaternary structure. 

## 4. Discussion

The results presented herein show that the study of tryptophan emission lifetime with phasors is a remarkable tool to assess protein self-assembly. With this method, we were able to determine a precise K_D_ value for the decamer–dimer equilibrium of a Prx1 class peroxiredoxin, a milestone in peroxiredoxin’s biophysical characterization.

Historically, SEC was the most commonly used approach to study the oligomerization of Prx1-class peroxiredoxins [33,34,35,36,37,38]. Although it is useful to study quaternary structure, it is not appropriate to use it to assess the equilibrium of dissociation. The issue with SEC is that the protein is continuously diluted during the measurements. Thus, the data obtained do not accurately represent the distribution of oligomeric forms (dimers and decamers) because the equilibrium is continuously shifted, leading to an overestimation of the C_0.5_. This was observed in our SEC experiments (Figure 5), as the shape of the decamer peaks exhibited tailing, even at high-protein concentrations above 10 μM. Additionally, the dissociation curve obtained from SEC data fitted rather poorly to the proposed model (R-Square = 0.89) and exhibited more than 50% error in K_D_ and C_0.5_ values. Moreover, the augmented baseline between peaks (Figure 5A) suggests either the presence of intermediate species, or the effect of sample dilution on the oligomerization equilibrium. The chromatographic traces can be fitted to multiple peaks (up to four); however, interpreting this as the existence of detectable populations of tetramers and hexamers is risky. The appearance of intermediate peaks is expected from the dissociation of decamers occurring during elution; if the concentration becomes sufficiently low and dissociation is faster than elution, dimers will appear within the tail of the decamer peak. 

On the other hand, the phasor analysis of hPrx1 fluorescence emission lifetime allowed us to unambiguously recognize hPrx1 decamers, dimers, and their mixture in the phasor plot. The data from lifetime phasors fitted significantly better to the model than SEC results (R-Square = 0.99), showing higher accuracy in the reported K_D_ and C_0.5_ values (around 15% error). Adding this to the fact that the phasor trajectories from decamers to dimers were linear (Figure 3), we can say that the oligomeric transition occurs without the formation of intermediate species under steady-state conditions. However, we cannot rule out the possibility of transient tetramer, hexamer, or octamer formation. 

As mentioned before, the fractions in the phasor plots corresponded to the fractional intensities of the emitted photons from decamers and dimers. Thus, changes in the quantum yield of the two species would affect the calculated molar fractions. It is worth mentioning that for hPrx1, we did not observe any change in the quantum yield of both oligomeric species (data not shown).

Surprisingly, the spectral phasors analysis was able to provide similar K_D_ and C_0.5_ values, despite an almost negligible change in the emission spectra. 

Small changes in the tryptophan environment can affect its lifetime and will thus result in a change in the corresponding phasor [14]. Both tryptophan residues of hPrx1 are part of the active site region. W86 is close to the dimer–dimer interface that is disrupted upon decamer dissociation, while W176 is farther from the interface (Figure 1C,D). The solvent accessible surface area (SASA) of W86 in the dimer and the decamer were identical (data not shown), ruling out the exposure of W86 to the solvent as the cause of the change in the emission lifetime of hPrx1. The active site of these Prxs is proposed to undergo unfolding during the catalytic cycle that is indispensable for enzymatic activity. It is hypothesized that a conformational equilibrium between a fully folded (FF) and a locally unfolded (LU) state exists and are associated with the stabilization or disruption of the decamer, respectively [27]. It is plausible that the FF-LU equilibrium depends on the oligomeric state of hPrx1 and that both W86 and 176 are sensing it, since they are part of the protein region that suffers the conformational change in the LU state. Therefore, it may be that the dimer is less conformationally constrained than the decamer, leading to an increase in the dynamics of the FF-LU transition, which is sensed by both tryptophans and is reflected as a change in the lifetime.

Peroxiredoxins are ubiquitous proteins and frequently very abundant [39]. The estimated intracellular concentration of hPrx1 is in the range of 6.8 to 120 µM (from reported values in different human cells [40]). On the basis of our result of C_0.5_ 1.36 µM, reduced hPrx1 in vivo would tend to be in the decamer state. However, there are multiple factors that may affect the oligomeric status of these peroxiredoxins (active site redox state, interaction partners, temperature, etc.); therefore, this C_0.5_ value should be used with caution since it may not be extrapolatable to all situations.

The fluorescence lifetime phasor analysis has shown the advantage of being sensitive, tag-free, and parsimonious in terms of amount of sample required. The method is versatile: one can readily test several conditions such as temperature, pressure, ionic strength, and pH with no practical or technical difficulties (in other methods, either it is not possible to test some of these conditions, or they become an arduous task). The measurements proved to be highly reproducible and rapid (the most time-consuming factor was sample incubation), and the data obtained fitted to our model accurately. As shown here, the results obtained for hPrx1 by phasors were validated by SEC experiments and also were proven to be of much better quality. Furthermore, C_0.5_ for hPrx1 determined by phasors agreed with previous results obtained by ITC (C_0.5_ = 1.3 μM [41]) and mass photometry (C_0.5_ *ca* 1 μM Figure S5 in [42]), which adds another layer of validation to the method.

## 5. Concluding Remarks

Fluorescence lifetime phasor analysis was found to be useful to study the dissociation of the decamer of hPrx1 and provided a K_D_ of 1.1 × 10^−24^ M^4^ and the corresponding C_0.5_ of 1.36 µM at 25 °C, pH 7.4, and 204 mM ionic strength. 

## Data Availability

Data are available upon request to the corresponding author.

## Abbreviations

K_D_, dissociation constant; hPrx1, human peroxiredoxin 1; τ, fluorescence emission lifetime.
